# *DKK3*’s protective role in prostate cancer is partly due to the modulation of immune-related pathways

**DOI:** 10.3389/fimmu.2023.978236

**Published:** 2023-02-09

**Authors:** Zainab Al Shareef, Mahmood Y. Hachim, Iman M. Talaat, Poorna Manasa Bhamidimarri, Mai Nidal Asad Ershaid, Burcu Yener Ilce, Thenmozhi Venkatachalam, Abdulla Eltayeb, Rifat Hamoudi, Ibrahim Y. Hachim

**Affiliations:** ^1^College of Medicine, University of Sharjah, Sharjah, United Arab Emirates; ^2^Sharjah Institute for Medical Research, University of Sharjah, Sharjah, United Arab Emirates; ^3^College of Medicine, Mohammed bin Rashid University of Medicine and Health Sciences, Dubai, United Arab Emirates; ^4^Pathology Department, Faculty of Medicine, Alexandria University, Alexandria, Egypt; ^5^Department of Physiology and Immunology, College of Medicine and Health Science, Khalifa University, Abu Dhabi, United Arab Emirates; ^6^Division of Surgery and Interventional Science, University College London, London, United Kingdom

**Keywords:** DKK3, prostate cancer, microenvironment, next-generation sequencing – NGS, tumor suppressor

## Abstract

While it is considered one of the most common cancers and the leading cause of death in men worldwide, prognostic stratification and treatment modalities are still limited for patients with prostate cancer (PCa). Recently, the introduction of genomic profiling and the use of new techniques like next-generation sequencing (NGS) in many cancers provide novel tools for the discovery of new molecular targets that might improve our understanding of the genomic aberrations in PCa and the discovery of novel prognostic and therapeutic targets. In this study, we investigated the possible mechanisms through which *Dickkopf-3* (DKK3) produces its possible protective role in PCa using NGS in both the DKK3 overexpression PCa cell line (PC3) model and our patient cohort consisting of nine PCa and five benign prostatic hyperplasia. Interestingly, our results have shown that DKK3 transfection-modulated genes are involved in the regulation of cell motility, senescence-associated secretory phenotype (SASP), and cytokine signaling in the immune system, as well as in the regulation of adaptive immune response. Further analysis of our NGS using our *in vitro* model revealed the presence of 36 differentially expressed genes (DEGs) between DKK3 transfected cells and PC3 empty vector. In addition, both *CP* and *ACE2* genes were differentially expressed not only between the transfected and empty groups but also between the transfected and Mock cells. The top common DEGs between the DKK3 overexpression cell line and our patient cohort are the following: *IL32, IRAK1, RIOK1, HIST1H2BB, SNORA31, AKR1B1*, *ACE2*, and *CP*. The upregulated genes including *IL32*, *HIST1H2BB*, and *SNORA31* showed tumor suppressor functions in various cancers including PCa. On the other hand, both *IRAK1* and *RIOK1* were downregulated and involved in tumor initiation, tumor progression, poor outcome, and radiotherapy resistance. Together, our results highlighted the possible role of the DKK3-related genes in protecting against PCa initiation and progression.

## Introduction

Prostate cancer (PCa) is considered one of the most common cancers and the leading cause of death among men worldwide (1–3). For decades, treatment modalities for this cancer were only limited to a few choices with hormonal therapy being considered the main therapeutic option (4). While several molecular targeted therapies were introduced in various cancers and led to improvement in the treatment landscape, no effective molecular therapies reach clinical use in PCa (4).

Genomic profiling of cancers using new techniques like next-generation sequencing (NGS) provides an important tool for discovering mutation profile and genomic aberrations that helps in the discovery of novel candidates for targeted therapy (5, 6).

Recent studies highlighted a protective and tumor suppressor role of stromal Dickkopf-3 (DKK3), a secreted glycoprotein that belongs to the DKK family of proteins in PCa (7, 8). Moreover, a study conducted by Al Shareef et al. showed that DKK3 expression is downregulated in PCa and its expression is associated with favorable outcomes (7). This action might be due to its opposing effect on the function of TGFBI and ECM-1; however, the mechanisms through which DKK3 might produce its anti-tumorigenic effect are not fully investigated.

Thus, this study aims to shed more light on the possible mechanisms through which DKK3 produces its protective effect in PCa and to discover novel genes and pathways that are modulated by DKK3. To achieve this, we explored the top differentially expressed genes (DEGs) in PCa cell lines with DKK3 overexpression *via* NGS. Moreover, using NGS, we further investigated the top DEG involving DKK3 expression in our patient cohort with PCa compared to patients with benign prostatic hyperplasia (BPH).

## Materials and methods

### Patient sample description and characteristics

This is a retrospective cohort study consisting of 14 prostate tissue samples (**Table 1**) from nine PCa patients from the Faculty of Medicine, Alexandria University, Egypt. The study was approved by the Research Ethics Committee of the Alexandria Faculty of Medicine. Five BPH specimens were retrieved from the University of Sharjah (UOS) tissue biobank after approval from the Al-Baraha Hospital Ethics Committee and the Ministry of Health and Prevention (MOHAP), the United Arab Emirates, following approval from the local Research Ethics Committee (approval reference number: REC-20-03-23-0, date 8 April 2020) and MOHAP (approval reference number: MOHAP/DXB-REC/JJA/No. 75/2020, date 30 August 2020).

**Table 1:** The clinicopathological parameters of our patient cohort.

Tissue characterization	No.	
**Total number of cases**	14	Prostate cancer (9) Benign prostatic hyperplasia (5)
**Age range (years)**	(56–94)	
**Median age**	67	
**Average age**	70	
Nationality		
Egyptian	9	
UAE	3	
Palestine	1	
Somalian	1	
Histological grade		
Low–moderate Gleason grade prostate cancer ≤3+4	5	5 (GL.7)
High Gleason grade prostate cancer >3+4	4	1 (GL.8) 2 (GL.9) 1 (GL.10)
**Lymphovascular/perineural invasion**	5	Lymphovascular and perineural invasion (3) Perineural invasion (1) Lymphovascular invasion (1)

### Transfection of DKK3 prostate cancer cell lines

Overexpression was performed using the GenEZ ORF clone of DKK3 constructed with pcDNA3.1+/C-(K)-DYK vector (OHu25813D-GenScript).

A total of 1 × 10^5^ cells per well in six-well plates were plated in the medium the day before transfection. The total volume of transfection complex (Opti-MEM medium, DNA, and ViaFect™ Transfection Reagent) added per well of a six-well plate was 100 μl. To the prewarmed 100 μl of serum-free medium (Opti-MEM), 1 μg of plasmid DNA (DKK3) was added and mixed. Since 3:1 ViaFect™ Transfection Reagent : DNA ratio was used, 3 μl of ViaFect™ Transfection Reagent was added. The ViaFect™ Transfection Reagent : DNA mixture was incubated for 20 min at room temperature. One hundred microliters of the ViaFect™ Transfection Reagent : DNA mixture was added per well drop by drop to the entire well of a six-well plate containing 2 ml of cells in the growth medium. The mixture was mixed gently by shaking very slowly and cells were returned to the incubator for 24–48 h. To compare the transfection efficiency, the cells of a six-well plate were kept non-treated as control, and the cells of a 6-well plate were treated only with the ViaFect™ Transfection Reagent without adding the DNA as a Mock.

After 24 h, transfected and non-transfected cells were collected by trypsinization for measuring the transfection efficiency by using QPCR and Western blot analysis.

For Western blot analysis, after the transfection, the cells were collected for protein extraction. Samples were separated on 10% SDS polyacrylamide gels. Each sample was loaded as 60 μg/well, probed with anti-DKK3 antibody (Ab187532), and developed with anti-rabbit IgG as a secondary antibody.

### Complementary DNA synthesis

Complementary DNA (cDNA) was synthesized from total RNA using a High-Capacity cDNA Reverse Transcription Kit (Applied Biosystems) according to the manufacturer’s instructions. Total RNA (150 ng) was mixed with 2 μl of 10x RT Buffer, 0.8 μl of 25x dNTP Mix, 2 μl of 10x RT Random Primer, 1 μl of reverse transcriptase, and nuclease-free water to make a final volume of 20 μl. The mixture was then incubated for 10 min at 25°C followed by 120 min at 37°C and 5 min at 85°C to inactivate the reverse transcriptase. The reactions were placed on ice for immediate use or at −20°C for long-term storage.

### Quantitative PCR

Gene expression was determined by quantitative PCR (qPCR) using 2x GoTaq qPCR Master Mix (Promega) and the QuantStudio3 Real-Time PCR thermal cycler (Applied Biosystems). Total RNA (150 ng) was reverse transcribed as described above, and 1 μl of cDNA was added to a reaction mix consisting of 5 μl of 2x GoTaq qPCR Master Mix, 5 μM of each forward and reverse primer, and nuclease-free water to make a final volume of 10 μl. PCR cycling conditions consisted of 50°C for 2 min, initial enzyme activation at 95°C for 10 min, denaturation at 95°C for 15 s, and annealing at 60°C for 1 min for 40 cycles. The expression levels of the target gene were normalized to an endogenous reference gene (18S), and the fold change, as a measure of relative expression, was calculated using the comparative Ct (2^–ΔΔCt^) method.

### Western blotting

For Western blot analysis, after the transfection, the cells are collected and lysed with M-PER Mammalian Protein Extraction Reagent (Thermo Scientific), supplemented with a protease inhibitor cocktail (Sigma) and DTT (Sigma). Protein (60 μg) was separated by 10% SDS polyacrylamide gel electrophoresis and transferred to a 0.45-μm nitrocellulose membrane (ThermoScientific). Membrane was incubated with anti-DKK3 antibody (Ab187532) and developed with anti-rabbit IgG as secondary antibody. β-actin antibody was used as control.

### Cell proliferation assay

The cellular viability of the cells was measured by the 3-(4,5-dimethyl thiazol-2-yl)-2,5-diphenyl tetrazolium bromide (MTT) proliferation assay after the 24-h transfection. Cells plated in flasks with 10% FCS RPMI and antibiotics were grown to 80% confluence before plating for proliferation assays. A total of 1 × 10^4^ cells per well were seeded in triplicate in 96-well plates and incubated at 37°C. The next day, transfection was done in 90 μl of media and 10 μl of transfection reagent mix including DNA was added. After 24-h incubation, 10 µl of MTT labeling reagent (5 mg/ml MTT) was mixed with the culture media without FBS and 100 μl was added to each well, which was then incubated in the dark for a further 4 h at 37°C. This step was followed by cell lysis with the addition of DMSO to dissolve formazan crystals. Spectrophotometric readings at A_560 nm_ were obtained on a GloMax (Promega). Each assay was carried out in triplicate and each experiment was repeated at least three times. Data are represented as the extent of cellular survival expressed as a percentage of control.

### Immunostaining

Four-micrometer BPH sections and PCa tissue sections were cut and placed onto positively charged slides (MIC3040, Scientific Laboratory Supplies) and polyethylene naphthalate (PEN) membrane glass slides (LCM0522, Applied Biosystems). For every sample, four unstained sections were prepared. The immunostaining was performed using the primary antibodies DKK3 1:300 dilution (Ab187532 rabbit monoclonal, Abcam), ECM-1 1:200 dilution (11521-1-AP rabbit monoclonal, Proteintech), TGFBI 1:200 dilution (Ab170874 rabbit monoclonal, Abcam), and PCK-1 1:200 dilution (MA182041 mouse monoclonal, Invitrogen). EnVision+ System-HRP-labeled polymer anti-rabbit (K400311, DAKO) and anti-mouse (K400111, DAKO) were used as secondary antibodies.

### Immunohistochemistry

Fourteen formalin-fixed paraffin-embedded (FFPE) BPA and PCa samples were each baked on a hot plate for 30 min at 60°C. The tissues were deparaffinized using two series of xylene (16446-2.5L-H, HONEYWELL) for 5 min each followed by rehydration in descending ethanol series, 100% ethanol, 90% ethanol, 70% ethanol, and 50% ethanol for 2 min each. Meanwhile, 1 L of antigen retrieval sodium citrate tribasic dihydrate buffer (71406-500G, SIGMA) was prepared at a pH of 6.0. The process of antigen retrieval was carefully carried out in a microwave oven for 15 min at 95°C. The retrieval buffer was allowed to cool at room temperature for 30 min. Slides were then washed under running tap water for 10 min and placed in a humidified rack. The tissue for staining was marked by a DAKO water-repellent pen (S2002, SIGMA). Then, 3% of hydrogen peroxide (7722-84-1, SDFine) was prepared, after which 100 μl was placed on the tissue and then washed with distilled water for 2 min. Prior to blocking with a protein blocking reagent (ab64264, Abcam), slides were washed with DPBS buffer (D8537-500ML, SIGMA) three times. The blocking solution was drained, and the primary antibody was added. The humidified rack was kept in a cold room overnight.

After 16 h, prior to the addition of secondary antibody, the slides were washed three times with DPBS for 3 min each. One hundred microliters of biotinylated secondary antibody was added to each slide and incubated for 30 min at room temperature in a humidified slide tray. After that, the slides were again washed with DPBS buffer for 3 min each. For the next steps, the HRP/DAB (ABC) Detection IHC kit (ab64264, Abcam) was used. One hundred microliters of HRP-labeled streptavidin peroxidase was added to each slide and incubated at room temperature for 20 min in a humidified slide tray, followed by washing three times with DBPS buffer for 3 min each. The DAB solution was prepared by adding DAB substrate up to 1 ml and adding a drop of chromogen. Subsequently, 100 μl of the prepared DAB solution was added to each slide and incubated for 4 min in a humidified slide tray. The slides were washed under running tap water for 5 min and then counterstained with hematoxylin stain (HEMM-OT-2.5L, BIOGNOST) for 2 min. The slides were then washed under running tap water for 5 min. After that, the tissues were rehydrated with four series of graded alcohol, 70% ethanol, 80% ethanol, 90% ethanol, and absolute ethanol by dipping slides for 5 min each. The slides were cleared in two series of xylene for 5 min each. The slides were then mounted with DPX (06522-100ML, SIGMA) mounting medium. Slides should be air-dried before microscopic examination. Images of the stained tissues were acquired with an Olympus BX43 microscope equipped with an Olympus DP75 camera with Cell Sens Entry Software.

### Laser capture microdissection

Target cell populations were microdissected using the Leica LMD 6 Laser Capture Microdissection System (Leica Microsystems CMS GmbH) equipped with Leica’s DFC7000 T camera. The cells were microdissected from immunostained BPH and PCa tissue sections adhered to PEN membrane slides (LCM0522, Applied Biosystems). The PEN membrane slides were loaded on the instrument with the tissue side down. For each sample, two sections adhered to the slide. The target area for every section was marked. The marked areas were collected for RNA extraction.

### Immunohistochemistry scoring

Fourteen patients in total, two slides per patient were stained for DKK3 (Ab187532, Abcam) and pan-cytokeratin (MA182041, Invitrogen).

Three randomized pictures were taken using a Leica DM750 microscope and scored based on the staining intensity as 0 (no staining), 1 (weak staining), 2 (moderate staining), or 3 (strong staining).

### Next-generation sequencing

RNA was extracted from the FFPE tissue sections using the RecoverALL nucleic acid extraction kit (Thermo Scientific, USA) using the manufacturer’s instructions. The extracted RNA samples were further purified and concentrated using the Zymo RNA clean and concentrator kit (Zymo Research, USA). Thus, purified RNA was quantified using qubit 3 fluorometers (Thermo Scientific, USA). using AmpliSeq Transcriptome (Thermo Fisher Scientific). In brief, ~50 ng of purified RNA was used for cDNA synthesis using the SuperScript VILO cDNA Synthesis kit (Invitrogen) followed by amplification using Ion AmpliSeq gene expression core panel primers. The enzymatic shearing was performed using FuPa reagent to obtain amplicons of ~200 bp, and the sheared amplicons were ligated with the adapter and the unique barcodes. The prepared library was purified using Agencourt AMPure XP beads (Beckman Coulter) and the purified library was quantified using the Ion Library TaqMan™ Quantitation Kit (Applied Biosystems). The libraries were further diluted to 100 pM and pooled equally with four individual samples per pool. The pooled libraries were amplified using emulsion PCR on Ion OneTouch™ 2 instrument (OT2) and the enrichment was performed on Ion OneTouch™ ES following the manufacturer’s instructions. Thus, prepared template libraries were then sequenced with an Ion S5 XL Semiconductor sequencer using the Ion 540™ Chip.

RNA-seq data were analyzed using the Ion Torrent Software Suite version 5.4. Alignment was carried out using the Torrent Mapping Alignment Program (TMAP). TMAP is optimized for Ion Torrent sequencing data for aligning the raw sequencing reads against the reference sequence derived from hg19 (GRCh37) assembly. To maintain specificity and sensitivity, TMAP implements a two-stage mapping approach. First, four alignment algorithms, BWA-short (BWA, http://bio-bwa.sourceforge.net), BWA-long, SSAHA (sanger.ac.UK), and Super-maximal Exact Matching, were employed to identify a list of candidate mapping locations. A further alignment process is performed using the Smith–Waterman algorithm (9) to find the final best mapping. Raw read counts of the targeted genes were performed using SAMtools (SAMtools view –c –F 4 –L bed file bam file). The quality control including the number of expressed transcripts is checked following the Fragments Per Kilobase Million (FPKM) normalization. DEG analysis was performed using R/Bioconductor package DESeq2 applied on raw read counts. Genes with less than 10 normalized read counts were excluded from further analysis.

### Bioinformatics analysis

#### DKK3 genetic alteration in prostate adenocarcinoma samples

To evaluate the rate of DKK3 mutation in a large cohort of PCa patients, we investigated DKK3 genetic alteration in prostate adenocarcinoma samples (Broad/Cornell, NAT Genet, 2012) through the publicly available database cBioPortal (http://www.cBioPortal.org/public-portal).

#### Identification of DEGs and pathway enrichment

To identify the differential expression of mRNAs between PC3 empty vector, Mock, and DKK3 transfected cells, RNASeq count files were uploaded to AltAnalyze—Comprehensive Transcriptome Analysis software. The hierarchical clustering in AltAnalyze is an important and useful method to filter specific pathways or genes and visualize expression patterns from databases. DEGs were selected if they showed >2 or <−2 log fold change with adjusted *p* < 0.1 between the two groups. Pathway enrichment was generated by the software.

## Results

### DKK3 is altered in PCa patients’ samples and its expression affects other genes’ expression

**Figure 1:**
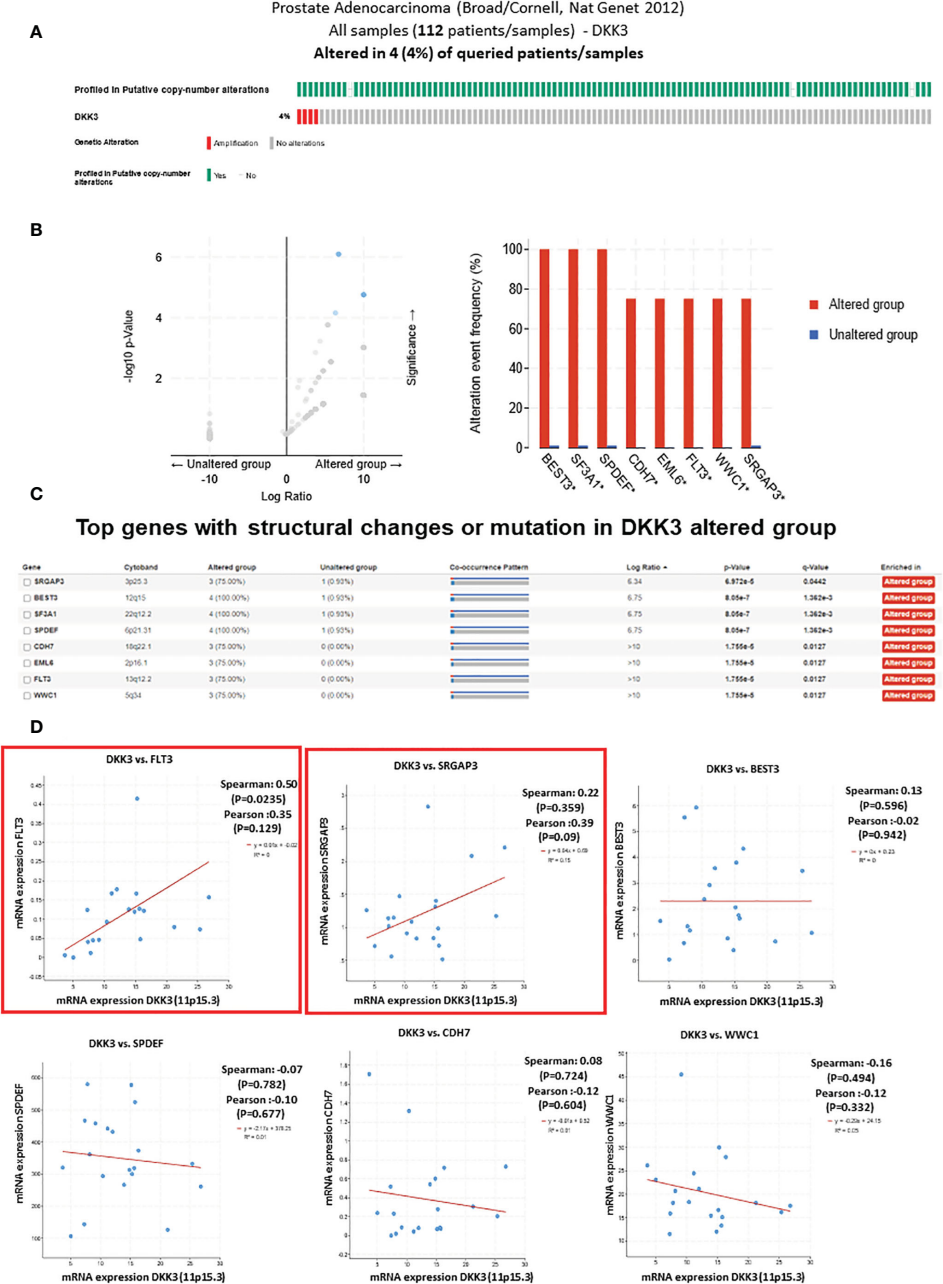
The genetic alterations of DKK3 and its correlation with other mutated genes in prostate adenocarcinoma samples obtained from TCGA, PanCancer Atlas database from cBioportal tool. **(A)** Oncoprint representation of alterations in DKK3 expression identified in Prostate Adenocarcinoma cohort (Broad/Cornell, Nat Genet 2012) obtained from TCGA, PanCancer Atlas database from cBioportal tool. **(B)** Volcano plot of differentially expressed genes in DKK3 altered and unaltered group (left panel). Top genes with the highest alteration frequencies in DKK3 altered group (right panel). **(C)** List of the top genes with structural changes or mutation in DKK3 altered group. **(D)** The mRNA of DKK3 and its correlation with the identified top mutated genes in DKK3 altered group.

Initially and to evaluate the rate of *DKK3* mutation in a large cohort of PCa patients, we investigated the rate of *DKK3* genetic alteration in prostate adenocarcinoma samples (Broad/Cornell, NAT Genet, 2012) through the publicly available database cBioPortal (http://www.cBioPortal.org/public-portal) (**Figures 1A–C**).

Our results showed amplification of the *DKK3* gene in 4% of prostate adenocarcinoma patients (**Figure 1A**). Interestingly, many genes were concomitantly altered in the DKK3 altered group. This includes *SRGAP3, BEST3, SF3A1, SPDEF, CDH7, EML6, FLT*s, and *WWC1* (**Figures 1B, C**).

Of interest, some of those genes were previously found to be deregulated in PCa including *SPDEF*, which functions as an androgen-independent transactivator of the prostate-specific antigen (PSA) promoter.

Next, we tried to further evaluate the association between *DKK3* and its concomitant altered genes. To achieve this, we investigated the correlation between DKK3 mRNA expression and the altered genes using the same database (**Figure 1D**). While most of the genes including *SPDEF, BEST3, CDH7*, and *WWC1* showed no significant association with DKK3 mRNA expression, our results showed a significant correlation between *DKK3* expression and both *FLT3* and *SRGAP3* expression.

### DKK3 overexpression promotes an anti-proliferative effect on prostate cancer cells

**Figure 2:**
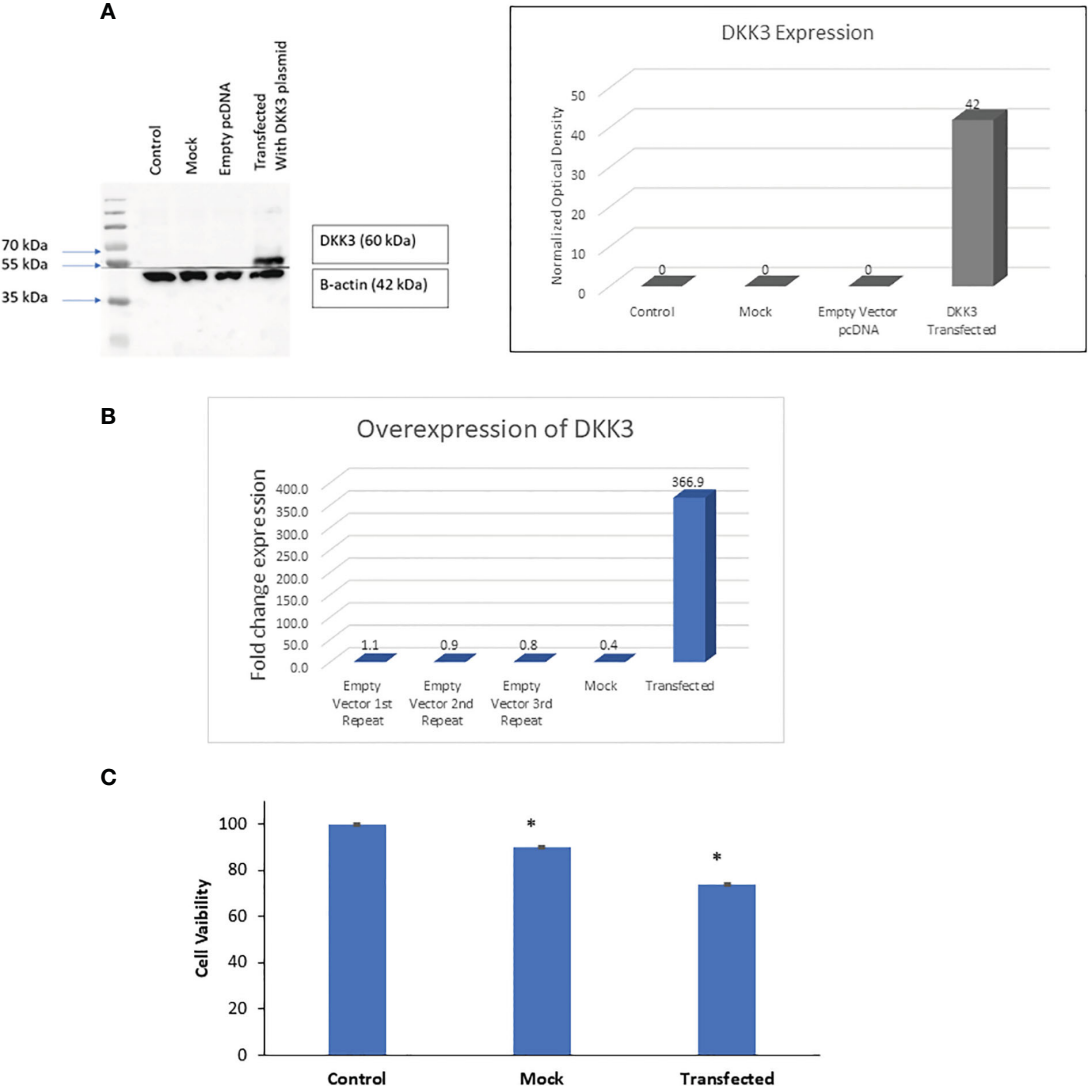
Effects of DKK3 overexpression on PC3 cell line viability. **(A)** Western blots of extracts from parental, MOCK, empty vector and DKK3 transfected PC3 cell line (Left panel). Densitometry analysis of DKK-3 normalized to beta-actin (Right panel). **(B)** Fold change expression of DKK3 expression in parental, MOCK, empty vector and DKK3 transfected PC3 cell line. **(C)** Cell viability of parental, MOCK, and Dkk3 transfected PC3 prostate cancer cell line, error bars show SD, Asterisk represents a significant difference (* p < .05).

Our previous work showed that loss of DKK3 expression in prostatic stromal cells and epithelial cells resulted in modulation of those cells’ behavior including increasing their proliferation and invasion capacity and modulation of *TGFBI* and *ECM-1* secretion. For that reason and to improve our understanding of the role of *DKK3* in PCa behavior, we overexpressed DKK3 expression in the PCa cell lines (PC3) in which *DKK3* mRNA expression is very low or not expressed (9) (**Figures 2A, B**). Our results demonstrated a significant overexpression of DKK3 protein expression in both transfected models. Next, we evaluate the biological effect of restoring DKK3 expression in the PC3 cell line. Our MTT assay showed that DKK3 overexpression in the PC3 cell line resulted in a significant reduction in the PC3 cell viability by approximately 26% compared to the control group (*p* = 0.01876) (**Figure 2C**). Indeed, and consistent with many studies (10), this indicates that restoring DKK3 expression in PCa cells inhibits their proliferation capacity.

### DKK3 overexpression upregulates the immune-related pathways in our *in vitro* model using the PC3 cell line

**Figure 3:**
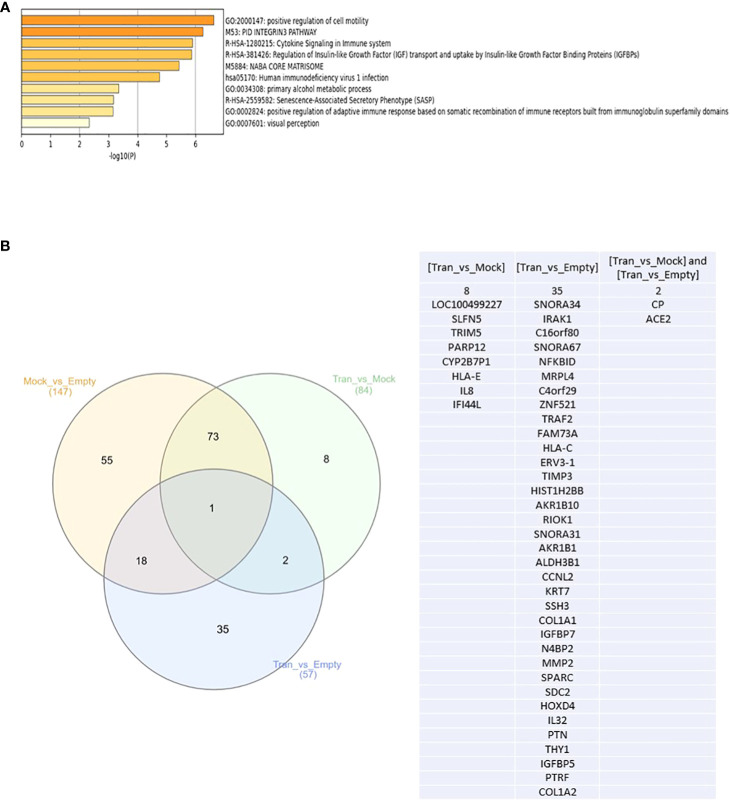
**(A)** Top enriched pathways in DKK3 transfected PC3 cell lines compared to empty vector and Mock cells obtained from our RNA seq analysis. **(B)** Intersecting the differential expressed genes between DKK3 transfected PC3 cell lines , empty vector and Mock cells obtained from our RNA seq analysis.

Next, and to evaluate if this change in PCa biological behavior is also associated with modification at the molecular level, we performed NGS and identified the differential expression of mRNAs between PC3 Empty vector, Mock, and DKK3 transfected cells. Our gene ontology analysis revealed significant modulation of genes involved in the regulation of cell motility, senescence-associated secretory phenotype (SASP), cytokine signaling in immune system, and regulation of adaptive immune response (**Figure 3A**).

Further analysis of our NGS analysis using our *in vitro* model revealed the presence of 35 genes that showed differential expression between DKK3 transfected cells and PC3 empty vector (**Figure 3B**), namely, *SNORA34, IRAK1, C16orf80, SNORA67, NFKBID, MRPL4, C4orf29, TRAF2, FAM73A, HLA-C, ERV3-1, TIMP3, HIST1H2BB, AKR1B10, RIOK1, SNORA31, ZNF521, AKR1B1, ALDH3B1, CCNL2, KRT7, SSH3, COL1A1, IGFBP7, N4BP2, MMP2, SPARC, SDC2, HOXD4, IL32, PTN, THY1, IGFBP5, PTRF*, and *COL1A2*. Our results showed *DKK3* to be the top upregulated gene in the DKK3 transfected cell line compared to empty vector, which clearly reflects the efficacy of our transfection model.

Other upregulated genes include *SNORA34, SNORA67, ZNF521, N4BP2, HLA-C, ERV3-1, TIMP3, HIST1H2BB, AKR1B10, SNORA31, AKR1B1, KRT7, COL1A1, IGFBP7, MMP2, SPARC, SDC2, IL32, PTN, THY1, IGFBP5, PTRF, PTRF*, and *COL1A2*.

In contrast, downregulated genes include the following: *IRAK1, C16orf80, NFKBID, MRPL4, C4orf29, TRAF2, FAM73A,SSH3, RIOK1, ALDH3B1, CCNL2*, and *HOXD4*.

Other genes were differentially expressed between DKK3 transfected and Mock cells. This includes eight genes: *LOC100499227, SLFN5, TRIM5, PARP12, CYP2B7P1, HLA-E, IL8*, and *IFI44L* (**Figure 3B**).

Two additional genes (*CP* and *ACE2*) showed differential expression not only between the transfected and empty group, but also between the transfected and Mock cells (**Figure 3B**).

### Laser capture microdissection approach to select heterogeneous DKK3 expression areas in benign and malignant samples from our patient cohort for next-generation sequencing

**Figure 4:**
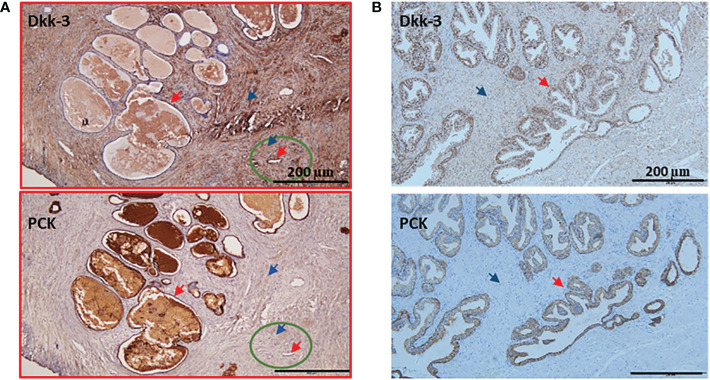
Examples of patient's IHC with lower expression of Dkk-3 in cancer compared to normal epithelium. **(A)** Gleason 8 prostate cancer patient was stained with Dkk-3 and Pan cytokeratin (PCK), the majority of the section shows a tumor (red frame), the normal adjacent tissue (green circle), the epithelium (red arrow), stroma (blue arrow). **(B)** Benning prostate hyperplasia patient was stained with Dkk-3 and Pan cytokeratin (PCK), the epithelium (red arrow), stroma (blue arrow), and scale bars 200 μm.

Next, and to investigate if the effect of DKK3 overexpression on the molecular profile of PCa cells that we observed *in vitro* was clinically relevant, we next performed RNAseq on biopsies obtained from our patient cohort that include patients with BPH and PCa patients (**Figure 4**). For the accurate selection of representative areas with low and high DKK3 expression in PCa and benign prostate hyperplasia patients (Ctrl), the tissues were stained with DKK3 as well as with anti-pan cytokeratin (PCK) to identify cancer epithelial and normal epithelial in a tissue section (**Figure 4**). We have followed the “quick-score” method that has been used in previous studies (7, 11); the intensity of staining was scored visually from 0 to 3: 0 (not stained), 1 (week stain), 2 (moderate stain), and 3 (strong stain). The average score of three randomly scored photos per patient section was taken. Furthermore, the tissue histopathological diagnosis was re-analyzed by our pathologist to confirm the patient’s Gleason scores and to demarcate the cancer tissue and normal adjacent tissue in the recruited sections.

An example of a patient’s “quick score” that shows DKK3 reverse expression and Gleason 8 patients’ section is shown in **Figure 4A**. The normal adjacent epithelium scored 2; however, it was reduced in the cancer epithelium to a score of 0. Normal adjacent stroma scored 1, and it increased in the cancer stroma to a score of 3. Importantly, a few patients were presaged with heterogeneous expression patterns. An example of BPH tissue (**Figure 4B**) section was scored as follows: epithelium (3), stroma (2).

**Figure 5:**
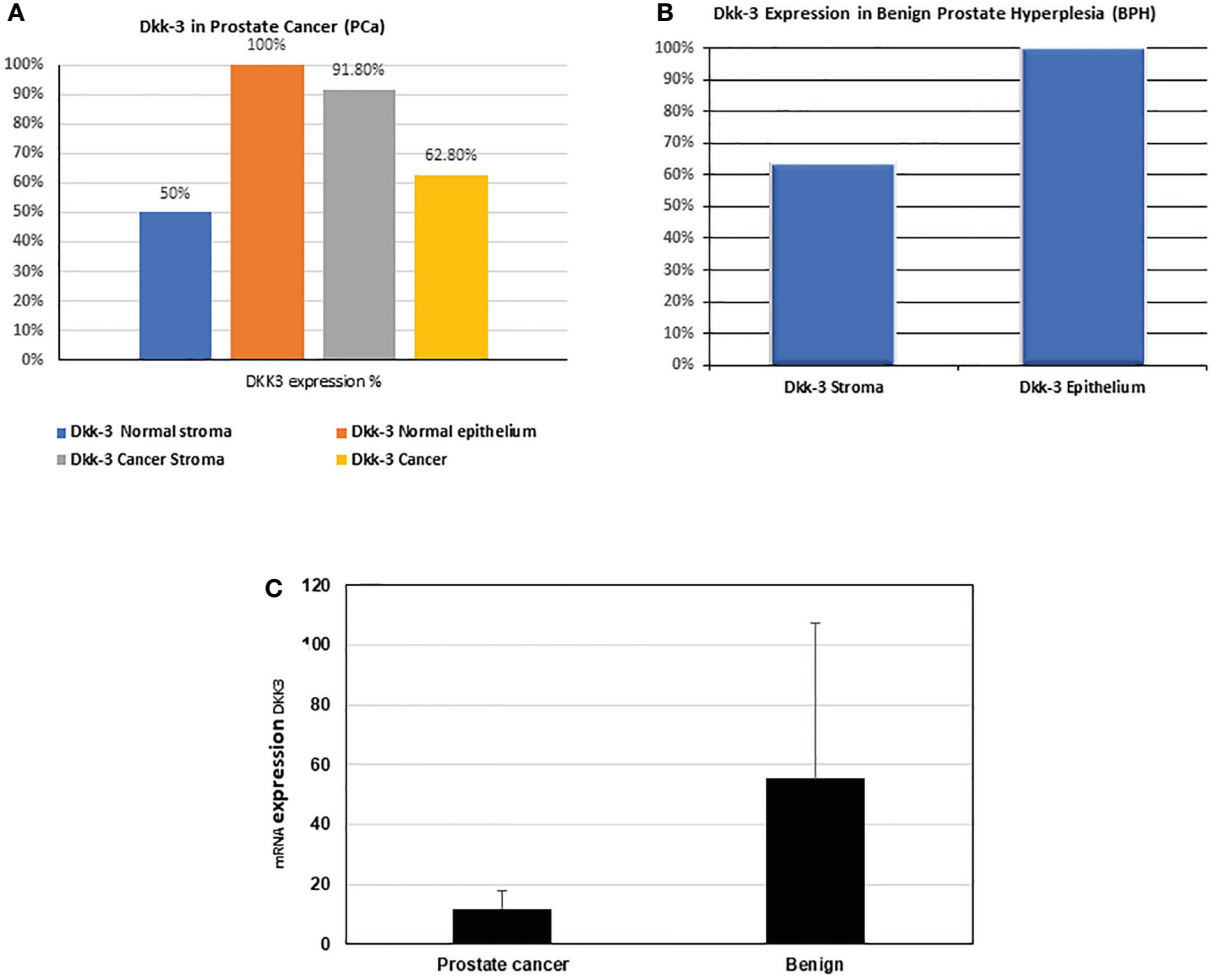
Percentage (%) of Dkk-3 expression in epithelium and stroma in our patients' cohort. **(A)** Dkk-3 expression in epithelium and stroma of cancer and normal adjacent tissue of nine PCa patients. **(B)** Dkk-3 expression in epithelium and stroma of being prostate hyperplasia tissue sections of five BPH patients. **(C)** The mRNA expression levels (from the RNAseq data) of DKK3 in the PCa patients compared with BPH.

In general, our results showed that DKK3 expression in cancer (**Figure 5A**) is reduced (62.8%) compared to normal adjacent epithelial (100%), which is consistent with the DKK3 pattern of expression demonstrated in European patients (7), apart from the higher expression of DKK3 in cancer stroma (91.8%) compared to normal adjacent stroma (50%), which is unique to the Middle Eastern patients and requires further elucidation with a higher sample size (**Figure 5A**). In BPH (**Figure 5B**), and similar to previous findings (12), higher expression of DKK3 in the epithelium (100%) was detected compared to stroma (63.64%).

**Figure 6:**
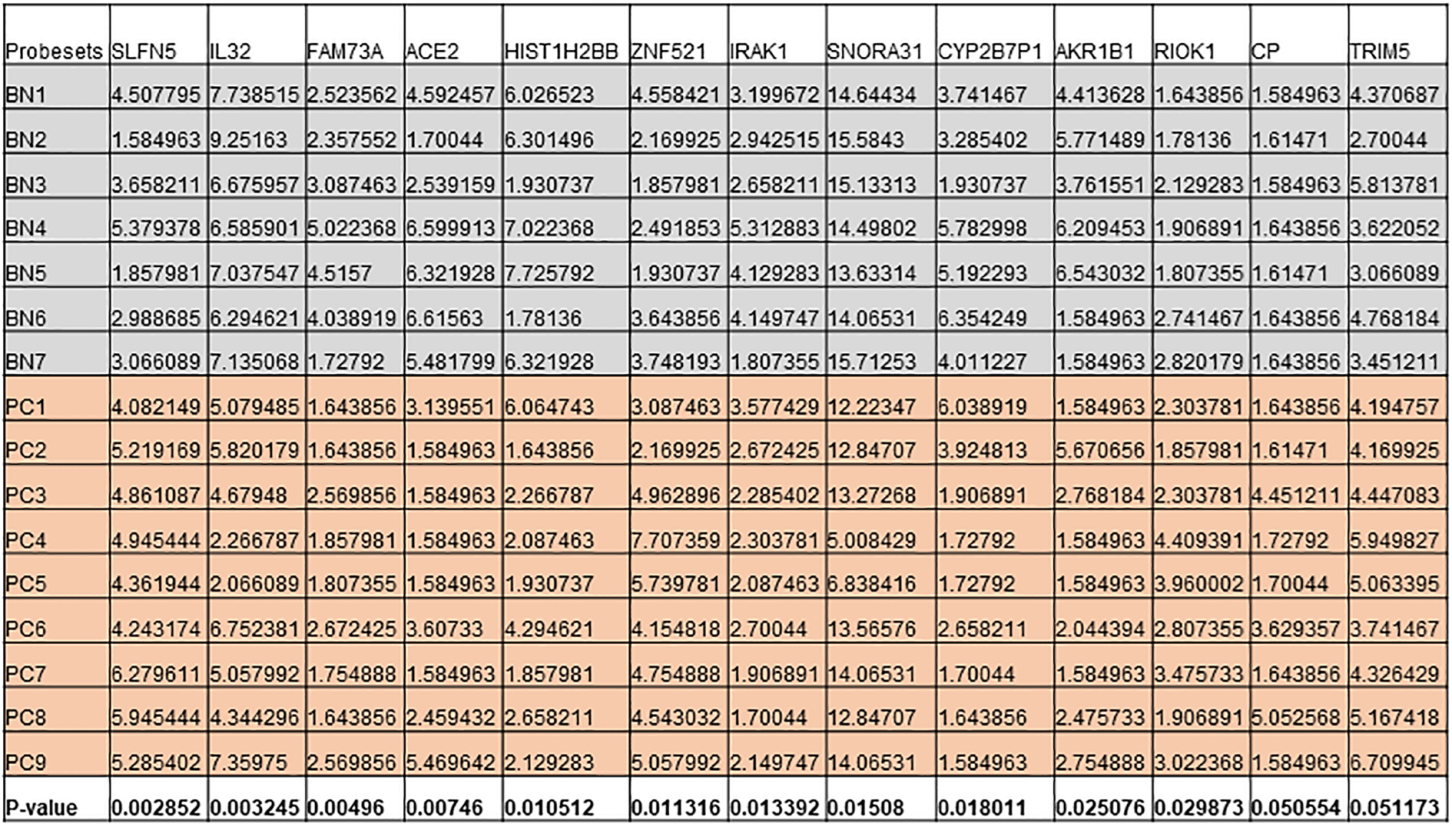
Expression levels of genes that are differentially expressed in both cell lines and patient cohort in Benign (BN) and Pca (PC).

Similarly, our results also showed that the mRNA expression of DKK3 was higher in BPH samples than in PCa samples (55.25 ± 52.26 compared to 11.89 ± 5.73) (**Figure 5C**).

### Identification of the key DEGs between the DKK;p overexpressed cell line and patient cohort

Next, we intersected the top DEGs between the DKK3 overexpressed cell line and patient cohort. Interestingly, our results showed a group of common genes that were significantly modulated in both groups (**Figure 6**). This includes *IL32, IRAK1, RIOK1, HIST1H2BB, SNORA31*, and *AKR1B1*, in addition to *ACE2 and CP*.

## Discussion

While several reports highlighted a tumor suppressor role of DKK3 and its downregulation in various tumors including PCa (7, 13, 14), there is no full understanding of the molecular mechanisms through which DKK3 might produce this anti-tumorigenic effect. In this study, we used a combined approach that includes *in silico* analysis and performing NGS to investigate the top DKK3 regulated genes using the DKK3 overexpression PC3 cell line, as well as our own patients’ samples.

Interestingly, our results showed that DKK3 overexpression in the PC3 cell line cause modulation of the group of pathways including genes involved in the regulation of cell motility, senescence-associated secretory phenotype (SASP), cytokine signaling in the immune system, and regulation of adaptive immune response.

While evidence about the link between DKK3 and immune response in cancers was sparse, few reports showed an immunomodulatory role and a possible therapeutic effect of REIC/DKK3 in some cancers through stimulation of the immune system (15). One possible mechanism through which DKK3 might affect the functions of immune cells is through the increment in the glucose level that will activate CD4+ T-cell proliferation and reduce its apoptotic levels (16). One of the reports suggested that the REIC/DKK3 stable cysteine-rich core domain is essential for the induction of DKK3-mediated anticancer immune responses (17).

Our results showed that *IL-32* is among the top DEGs that are upregulated in the DKK3 transfected PC3 cell line compared to empty vector in the PC3 cell line and, at the same time, its expression was downregulated in our PCa samples compared to samples from patients with BPH. While its role in some cancers was controversial, several reports highlighted a possible role of IL-32 in inhibiting cell proliferation in colon, prostate, and melanoma malignant cells (18, 19). This effect was suggested to be mediated through lymphocytes, dendritic cells, and other cytokines (18, 19). Additional mechanisms explained the IL-32 anti-tumor activities through its ability to modulate other cytokines and immune cells including TNF-α (20).

Another gene that was upregulated in the DKK3 transfected PC3 cell line compared to the empty vector and confirmed to be upregulated in BPH samples compared to PCa samples is *HIST1H2BB* (Histone Cluster 1 H2B Family Member B), which is a protein-coding gene, recently described as a tumor suppressor gene (21).

Other genes that were also shown to be upregulated in DKK3 transfected cells and in samples obtained from benign lesions compared to malignant tissues were *SNORA31* and *AKR1B1*. Interestingly, *SNORA31* belongs to the small nucleolar RNAs (snoRNAs) family. While those genes were considered housekeeping genes with no specific function in cancer biology, recent reports showed that snoRNA expression is modulated during cancer progression (22). A recent study that investigated several snoRNAs in the metastatic vs. non‐metastatic xenografts, as well as clinical databases showed SNORA31 to be the only snoRNA that was downregulated in metastatic compared to non-metastatic xenografts. This finding was further confirmed in clinical data that showed *SNORA31* is downregulated in metastatic samples compared to normal samples (22).

In contrast to the DKK3 upregulated genes, our results showed a group of genes that were downregulated in DKK3, and some of them were confirmed to be differentially expressed in our patient cohort of PCa samples and samples from BPH patients.

*RIOK1* was one of those genes that were downregulated in DKK3 transfected cells, and its expression was found to be upregulated in PCa samples compared to BPH cases. This gene belongs to the *RIO* (right open reading frame) family and consists of atypical protein kinases (23). RIOK1 is overexpressed in some cancers including colorectal and lung cancer. Moreover, RIOK1 was found to promote tumor growth as well as malignant cell invasive behavior (24, 25).

Interleukin-1 receptor associated kinase 1 (*IRAK1*) was another interesting gene that was downregulated in DKK3 transfected cells and was differentially expressed between benign and malignant samples from our patient cohort. Interestingly, IRAK1 is an essential component in the Myddosome complex and is considered one of the TLR signaling main effectors (26). Interestingly, IRAK1 is also overexpressed in several cancers including liver, lung, breast, and endometrium; moreover, its expression was associated with unfavorable outcomes and poor survival (26–28). Moreover, IRAK1 was found to be responsible for radiotherapy resistance in human cancer cells; for that reason, selective IRAK1 inhibitors were proposed for cancer treatment (28).

Our finding that showed high expression of DKK3 in cancer stroma compared to normal adjacent tissue, which is unique to the Middle Eastern patients, might need further elucidation with a higher patient sample size. One of the explanations for this finding is the fact that most of our patients (55.55%) were low–moderate Gleason grade PCa and previous reports found a negative association between DKK3 expression and tumor grade in various cancers (14, 29).

In summary, our study highlighted possible novel mechanisms and genes that are modulated by DKK3 that might help in improving our understanding of its tumor suppressor and protective role in PCa. The use of a combined approach to obtain the final short-listed genes in both the cell line model and the patient cohort might help in minimizing the effect of transfection or any other factor used in the *in vitro* model and assure the clinical significance of our findings.

## Data availability statement

The data presented in the study are deposited in figshare: https://figshare.com, the raw data can be accessed from the following link: https://figshare.com/articles/dataset/DKK_protective_role_in_prostate_cancer_is_partly_due_to_the_modulation_of_immune-related_pathways/21657629.

## Ethics statement

The studies involving human participants were reviewed and approved by Research Ethics Committee (approval reference number: REC-20-03-23-0, date 08/04/2020) and MOHAP (approval reference number: MOHAP/DXB-REC/JJA/No. 75/2020, dated 30/08/2020). Written informed consent for participation was not required for this study in accordance with the national legislation and the institutional requirements.

## Author contributions

ZS and IH: Conceptualization, data curation, investigation, methodology, writing—original draft preparation, writing—review and editing. RH: NGS supervision, analysis and writing, and review and editing. IT: Samples collection, histopathological diagnosis, IHC interpretation, and draft review and editing. MH: Bioinformatics and writing—review and Editing. ME, BI, TV, PB, and AE: Performing experiments and data analysis. All authors contributed to the article and approved the submitted version.

## Supplementary material

Supplementary Table 1 RNASeq data of cell lines as well as patient cohorts with the respective adj-*p*-value and fold changes for each gene in each condition.
